# Supplementary material for: Quantifying uncertainty in wave attenuation by mangroves to inform coastal green belt policies
Source: Commun Earth Environ. 2025 Apr 3;6(1):258. doi: 10.1038/s43247-025-02178-4 (PMC11968402; doi:10.1038/s43247-025-02178-4)
Supplement: Supplementary file 2 — Supplementary material [file 43247_2025_2178_MOESM2_ESM.pdf]

Supplementary materials

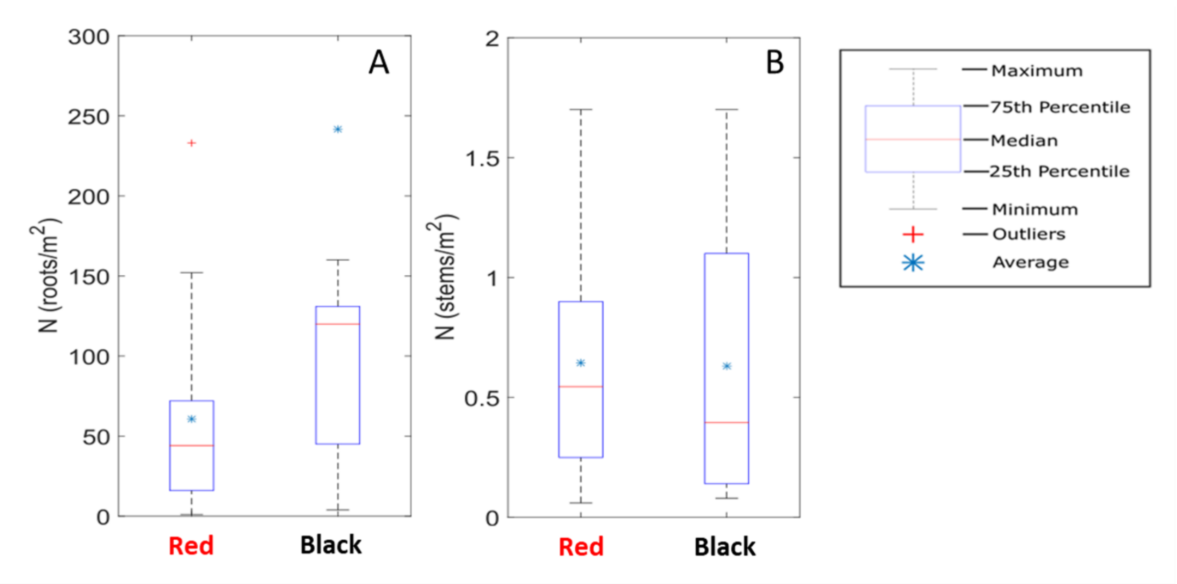

S1. Number of roots and stems per square meter for black and red mangroves. Data derived from the following sources: Mazda et al. (1997), Cole et al. (1999), Krauss et al. (2003), Brinkman (2006), Narayan (2009), Husrin et al. (2012), Tusinski (2012), Bo (2012), Xiaofeng (2014), Horstman et al. (2014).

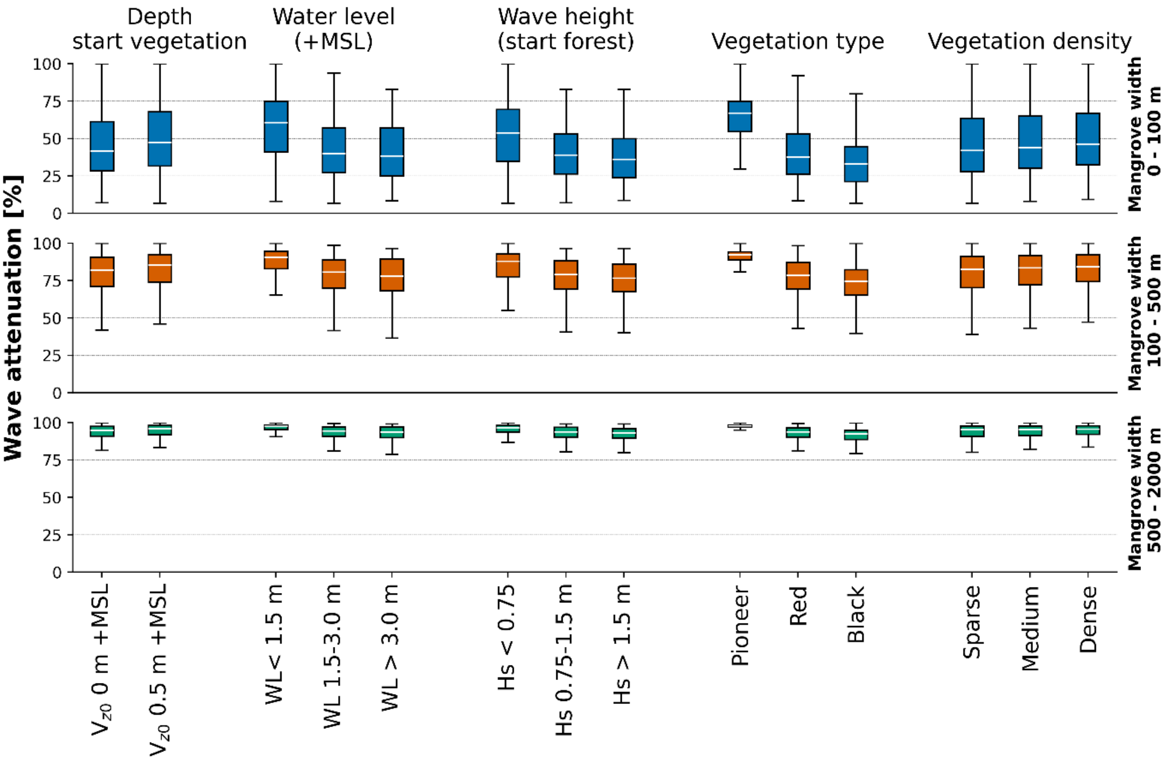

S2. Contribution of tested parameters to wave attenuation with on the y-axis wave attenuation percentage and on the x-axis (1) starting depth of the vegetation (Vz0), (2) water level (WL), (3) wave height (Hs), (4) vegetation type, and (5) vegetation density for three different mangrove width classes (0-100, 100-500 and 500-2000 meter).
